# Cholesterol Transport in Wild-Type NPC1 and P691S: Molecular Dynamics Simulations Reveal Changes in Dynamical Behavior

**DOI:** 10.3390/ijms21082962

**Published:** 2020-04-22

**Authors:** Nadia Elghobashi-Meinhardt

**Affiliations:** Department of Physical and Theoretical Chemistry, Technische Universität Berlin, 10623 Berlin, Germany; n.elghobashi-meinhardt@campus.tu-berlin.de; Tel.: +49-30-314-79386

**Keywords:** Niemann–Pick C1 (NPC) protein, cholesterol, P691 mutant, itraconazole binding site (IBS), molecular dynamics (MD) simulations

## Abstract

The Niemann–Pick C1 (NPC1) protein is the main protein involved in NPC disease, a fatal lysosomal lipid storage disease. NPC1, containing 1278 amino acids, is comprised of three lumenal domains (N-terminal, middle lumenal, C-terminal) and a transmembrane (TM) domain that contains a five helix bundle referred to as the sterol-sensing domain (SSD). The exact purpose of the SSD is not known, but it is believed that the SSD may bind cholesterol, either as a part of the lipid trafficking pathway or as part of a signaling mechanism. A recent cryo-EM structure has revealed an itraconazole binding site (IBS) in the SSD of human NPC1. Using this structural data, we constructed a model of cholesterol-bound wild-type (WT) and mutant P691S and performed molecular dynamics (MD) simulations of each cholesterol-bound protein. For WT NPC1, cholesterol migrates laterally, in the direction of the lipid bilayer. In the case of P691S, cholesterol is observed for the first time to migrate away from the SSD toward the N-terminal domain via a putative tunnel that connects the IBS with the lumenal domains. Structural features of the IBS are analyzed to identify the causes for different dynamical behavior between cholesterol-bound WT and cholesterol-bound P691S. The side chain of Ser691 in the P691S mutant introduces a hydrogen bond network that is not present in the WT protein. This change is likely responsible for the altered dynamical behavior observed in the P691S mutant and helps explain the disrupted cholesterol trafficking behavior observed in experiments.

## 1. Introduction

The Niemann–Pick C1 (NPC1) is a 140 kDa lysosomal protein (1278 amino acids) that is one of the central biochemical players in NPC disease, a fatal, autorecessive, lysosomal lipid storage disease [1]. The past two decades have witnessed considerable progress in understanding the workings of this lysosomal protein (see the review in [2]). It is believed that after endocytosis, low-density-lipoprotein (LDL)-derived cholesterol is bound by the smaller, soluble NPC2 protein (25 kDa, 131 amino acids) in the lumen and transferred to NPC1, after which the cholesterol is further processed out of the lysosome [3,4]. The co-crystal structure (2.4 Å) of NPC1 with the smaller, soluble NPC2 protein provided insight into how NPC1 and NPC2 may form a stable protein-protein complex [5]. With the continuing improvement in cryo-electron microscopy (EM) imaging technology, high-resolution structures of full-length NPC1 have emerged that have provided the community with further insight into the architecture of this large protein and the possible significance of mutations in specific domains [6,7].

Mutations in either NPC1 or NPC2 may lead to NPC disease, with 95% of mutations mapping to the larger, membrane-bound NPC1 [1]. How specific mutations in NPC1 are related to disrupted cholesterol trafficking and ultimately to disease phenotype is not understood. Applying machine learning techniques, Wang et al. recently analyzed the structural determinants to uncover connections between epigenetic factors and disease phenotype [8]. Indeed, some mutants, e.g., homozygous I1061T, give rise to a severe disease phenotype with abolished cholesterol trafficking, while others are related to a mild disease phenotype [9,10].

Interestingly, NPC1 is one of many proteins that contains a conserved five transmembrane (TM) (amino acids 621–797) bundle referred to as the sterol-sensing domain (SSD) that is responsible for a range of trafficking and signaling processes [5,11,12,13]. The SSD demonstrates a high degree of homology with the SSD of other proteins including HMG-CoA reductase (HMGR), the rate-controlling enzyme in cholesterol synthesis, PATCHED1 (PTCH1), a signal transducer for the Hedgehog pathway, and SCAP, a cleavage-activating protein associated with sterol regulatory element binding proteins (SREBPs) [14]. The role of the SSD in NPC1 is not understood, but it is believed that it is critical for sterol metabolism. Especially the recent elucidation of atomic-resolution structures of PTCH1, belonging to the same resistance-nodulation-division (RND) superfamily as NPC1 [15], has raised the question of whether NPC1 evolved to rely on the SSD in a similar manner as PTCH1, namely as a cholesterol sensor [16]. Indeed, the cryo-EM structure of PTCH1 shows two sterol molecules, one located in the cavity between the two extracellular domains and the second located adjacent to the sterol-sensing domain [17].

**Figure 1:**
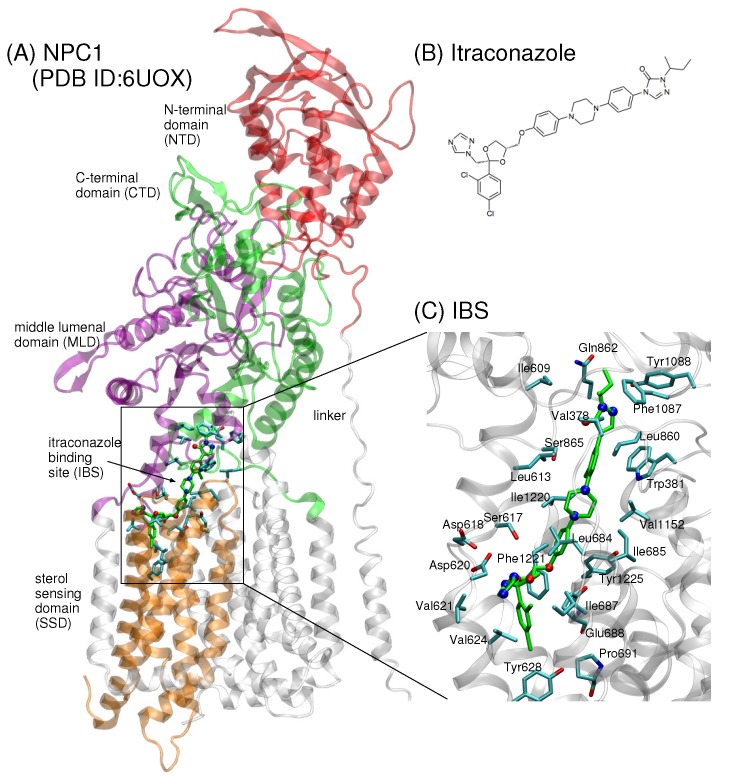
(**A**) The cryo-EM structure of human NPC1 bound to itraconazole (PDB ID 6UOX, 4.0 Å) shows the small molecule localized at the interface between the sterol-sensing domain (SSD, aa 621–797), the middle lumenal domain (MLD, aa 370–621), and the C-terminal domain (CTD, aa 854–1083) of NPC1 [18]. (**B**) The chemical structure of the small molecule itraconazole. (**C**) Up-close view of itraconazole (shown in green stick representation, oxygen atoms in red and nitrogen atoms in blue). Side chains of amino acids within a 5 Å radius from itraconazole atoms are depicted in blue stick representation with oxygen atoms in red.

Recently, a 4.0 Å cryo-EM structure of human NPC1 was published (PDB ID 6UOX) showing itraconazole bound in NPC1 at the juncture between the transmembrane domain (TMD) helices, middle lumenal domain (MLD, aa 370–621), and C-terminal domain (CTD, aa 854–1083) (Figure 1A,B) [18]. A small molecule belonging to a class of antifungal drugs, itraconazole is known to inhibit cholesterol export from lysosomes [19]. The position of the binding site in [18] is one of five binding sites previously identified computationally [20] and near a putative tunnel that extends 120 Å through the center of the protein, also in agreement with a previously predicted transfer path [20]. The itraconazole binding site (IBS) is reminiscent of the palmitate binding site in PTCH1 [16,21]. Enzymatic assays further demonstrate that itraconazole blocks the putative sterol tunnel and thereby inhibits NPC1 activity [18]. Intriguingly, a similar tunnel was recently identified in the analogous NPC1 protein in yeast, NCR1, that shares 32% sequence identity with human NPC1 [13].

**Figure 2:**
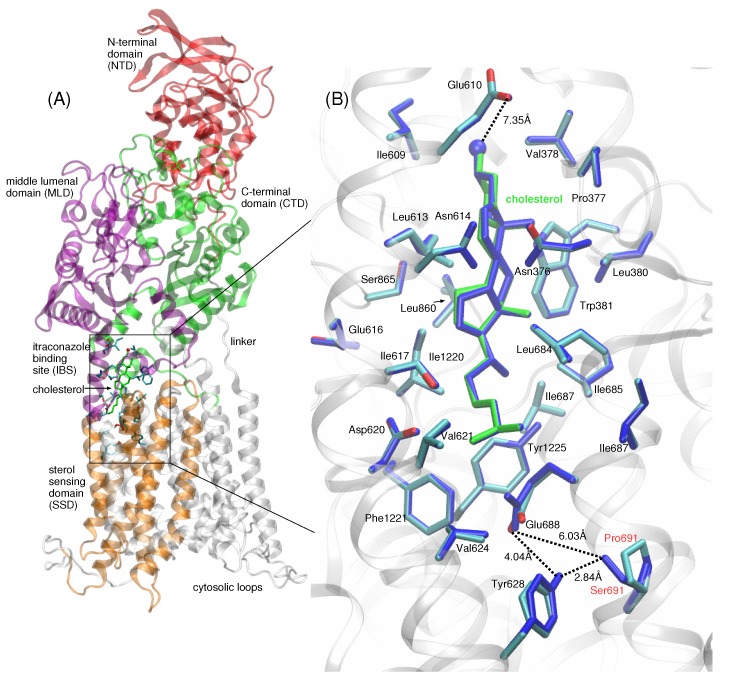
(**A**) Full-length NPC1 is shown in ribbon representation, with the N-terminal domain (NTD, aa 25–264) shown in red, MLD (aa 370–621) in purple, CTD (aa 854–1083) in green, and the five helices of the TMD comprising the SSD (aa 621–797) shown in orange. The cholesterol molecule (green stick representation) is located at the itraconazole binding site (IBS), with cholesterol’s isooctyl tail pointing downward relative to the N-terminal domain (NTD) of NPC1. Side chains of amino acids within a 5 Å radius from cholesterol atoms are depicted in blue stick representation. The majority of the amino acid side chains lining the IBS are hydrophobic. (**B**) Up-close view of cholesterol (shown in green stick representation) in the IBS of WT (multi-colored) and P691S (dark blue). The hydroxyl group points toward the side chain of Glu610 (∼7.35 Å separation between hydroxyl oxygen and Glu610 O_*γ*_). At the base of the IBS, the side chain of Glu688 (proton not shown) interacts with the side chains of Ser691 in P691S and Tyr628; in WT NPC1 (Pro691), this interaction is absent. Dotted lines are drawn to indicate the separation between Glu688 and P691 (S691) side chain atoms, as well as the relative position of cholesterol in the IBS; the dotted lines do not indicate hydrogen bonds. Distances are given in Å.

The significance of a putative tunnel is still not known, but it has been suggested that LDL-derived cholesterol may be transported via such a tunnel from the lumen to the lysosomal membrane [18]. The fact that itraconazole binding blocks the putative sterol tunnel, thereby inhibiting NPC1 activity, suggests that the structural features of the IBS are critical for the cholesterol transport. One amino acid implicated in this process is Pro691, located at the base of the transport tunnel (Figure 2B). Pro691 is conserved in NPC1, SCAP, PTCH1, and HMG-CoA, and it is believed to be critical for direct binding of cholesterol in the SSD [22]. The P691S mutant in human NPC1 is delivered to lysosomes, but demonstrates defective cholesterol trafficking [22,23]. In the recent work of Long et al., the authors raised the question of whether the P691S mutation may disrupt the hydrophobic environment at the base of the IBS, thereby obstructing the exit passage of cholesterol to the lysosomal membrane [18].

To address this question, here we model a cholesterol molecule in the same position in WT and the P691S mutant and perform molecular dynamics (MD) simulations of full-length NPC1 in the fully-solvated lipid bilayer.

## 2. Results

The position of cholesterol modeled in the IBS of WT NPC1 and P691S is shown in Figure 2A,B. The IBS is located at the interface between the SSD (aa 621–797, shown in orange in Figure 2A) and MLD (purple) and CTD (green). Based on the structure of Long et al. [18], we modeled the hydroxyl group of the cholesterol molecule pointing towards the lumenal domains with the isooctyl tail pointing in the opposite direction. The hydroxyl group points toward the side chain of Glu610 (∼7.35 Å separation between the hydroxyl oxygen and Glu610 O_*γ*_) (Figure 2B). The majority of the amino acid side chains lining the IBS are hydrophobic. At the base of the IBS, the side chain of Glu688 (proton not shown) interacts with the side chains of Ser691 in P691S and Tyr628; in WT NPC1 (Pro691), this interaction is absent (see the up-close view of the interactions in Figure 2B; hydrogen bonding). In a previous study, the pKa of Glu688 was determined to be ∼10, so within the acidic lysosome (∼pH 5.5), the side chain is likely protonated and is modeled as such in our study [20].

**Figure 3:**
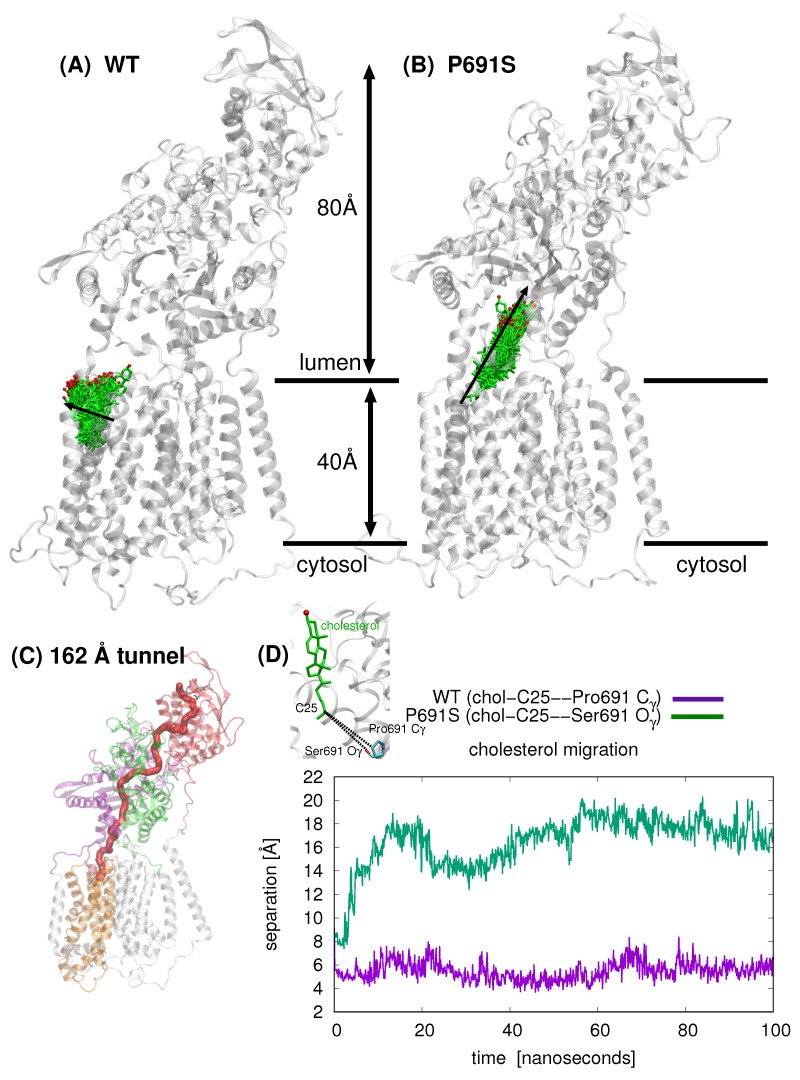
Comparison of cholesterol position (cholesterol shown in green stick representation with the hydroxyl oxygen atom in red) over 100 ns simulation time for (**A**) WT NPC1 and B) P691S (NPC1 rotated in (**B**) to allow for better visualization of cholesterol passage). For WT NPC1, cholesterol motion is lateral (indicated with black arrow) in the direction of the lipid bilayer (bilayer not depicted), whereas in the case of P691S, the cholesterol molecule migrates along the putative tunnel that runs through the junction between the MLD and CTD. (**C**) The tunnel, 162 Å in length (computed with MoleOnline [24] using a cholesterol-free WT NPC1 model; details in Section 4) in NPC1 is depicted in red. (**D**) Comparison of cholesterol position for WT (purple) and P691 (green) over the course of 100 ns MD. The separation (Å) between the cholesterol C25 atom and Pro691-side chain C_*γ*_ and Ser691 O_*γ*_ atoms, respectively, are plotted as a function of time (ns).

Snapshots taken from 100 ns MD simulation for cholesterol-bound WT and cholesterol-bound P691S are shown in Figure 3A,B, respectively; the position of cholesterol is shown after each nanosecond (100 snapshots). The overall direction of sterol motion differs for WT and P691S (indicated with black arrows in Figure 3A,B). In Figure 3C, the cholesterol position is shown as the separation between the C25 atom of cholesterol and Pro691-side chain C_*γ*_ and Ser691 O_*γ*_ atoms, respectively. The striking difference in dynamical behavior can be described for WT NPC1 as a lateral movement of cholesterol toward the lipid bilayer, whereas in P691S, the cholesterol molecule travels upward toward the NTD, translocating nearly 10 Å over the 100 ns simulation time, via the putative transport tunnel previously predicted [18,20]. Importantly, the cholesterol motion in P691S follows a cholesterol flow path recently established using spatial covariance analyses [8].

**Figure 4:**
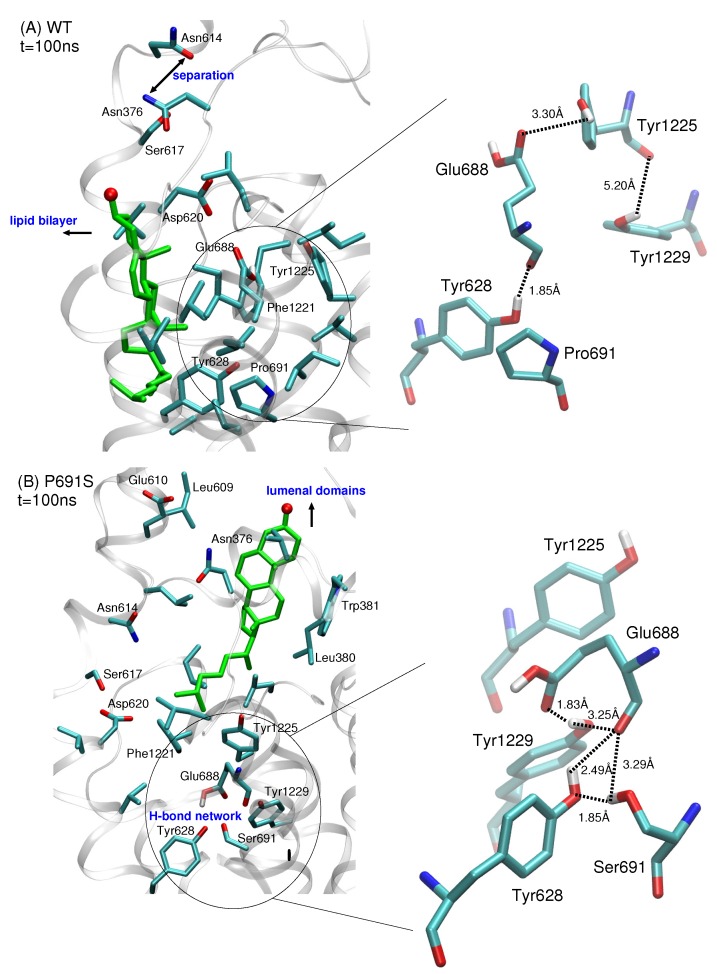
Comparison of IBS side chain interactions for (**A**) WT NPC1 and (**B**) P691S for snapshots taken at t = 100 ns from MD simulations. Amino acid side chains within 3 Å of cholesterol are shown; cholesterol is shown in green stick representation with the hydroxyl oxygen atom in red. In both simulations, the separation of helices containing Asn376 and Asn614 accompanies the movement of sterol. (**A**) In WT NPC1, the side chain of Glu688 points upward toward the lumenal domains and interacts with the side chain of Tyr1225, while Pro691 is surrounded predominantly by hydrophobic side chains. (**B**) In P691S, the protonated side chain of Glu688 reorients itself, recruiting amino acids Tyr628, Ser691, and Tyr1229 with which it forms a hydrogen bonding network. Dotted lines are drawn to indicate the separation between side chain atoms and do not imply hydrogen bonds for separations greater than 3.5 Å. All distances are given in Å.

We analyzed the structural features of the IBS to understand why the dynamical behavior of cholesterol differs when proline at position 691 is mutated to serine. In both WT and P691S, a separation of the salt bridge interactions between side chains of Asn614 and Asn376 is observed. In WT NPC1, the movement of the protein helices containing these Asn residues allows cholesterol to translate toward the bilayer. The protonated side chain of Glu688 points toward the lumenal domains and forms a hydrogen bond with the hydroxyl group of Tyr1225 (Figure 4A). Pro691 is located in a predominantly hydrophobic pocket.

In P691S, on the other hand, the side chain of Glu688 reorients itself toward Ser691. This reorientation is critical as it further recruits the side chains of Tyr628 and Tyr1229, enabling the formation of a hydrogen bonded network (Figure 4B). This hydrogen bonded network is maintained throughout the 100 ns simulation. Importantly, Ser691 also interacts strongly with the backbone oxygen atom of Glu688. The reorientation of Glu688 allows for subsequent conformational changes that are instrumental in both preventing lateral translocation of cholesterol and in producing the putative sterol transport tunnel.

**Figure 5:**
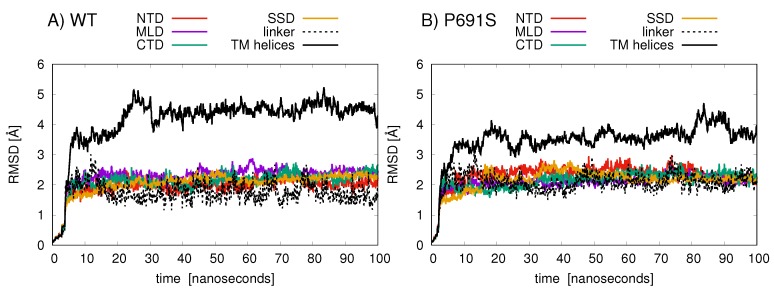
The RMSD (Å) values of amino acid backbone atoms are plotted for the various NPC1 structural domains over the 100 ns simulation for (**A**) cholesterol-bound WT and (**B**) cholesterol-bound P691S. The domains are colored as follows: NTD (amino acids 30–250, red); linker (amino acids 251–259, dashed black); MLD (amino acids 374–620, purple); TM helices (amino acids 260–373, 621–797, and 1084–1278, black solid line); SSD (amino acids 621–797, orange); CTD (amino acids 861–1083, green).

Root-mean-squared-deviation (RMSD) analyses of protein backbone atomic positions over the 100 ns MD simulations indicate that the TMD helices in WT undergo larger conformational changes than do the helices in P691S (Figure 5). The NTD, MLD, and CTD exhibit relatively conserved backbone motion, with RMSD values between 2 and 3 Å. The TMD helices in WT (aa 260–373, 621–797, 1084–1278) show the largest RMSD changes (black line in Figure 5A), while for P691S, these values are somewhat lower (black line in Figure 5B).

To check the correlation between the protein domains, distance correlation coefficients (DiCC) (Table 1) for each of the protein domains of WT and P691S NPC1 were calculated from the simulated trajectories of cholesterol-bound NPC1 (see Section 4 for details). For highly correlated motion between protein domains, the DiCC are close to 1.00, whereas for uncorrelated motion, the DiCC are close to 0.00. In WT (Table 1), the NTD and CTD show the highest correlation, whereas for P691S (Table 1), the correlations are highest between NTD and MLD. In WT NPC1, the correlation between the TMD and the NTD is around 0.9, whereas in P691S, this correlation drops to ∼0.8, suggesting a significant disruption in long-range communication between domains. The largest changes in DiCC are observed for correlations between CTD and cytosolic loops (ΔDiCC = 0.209), followed by correlations between NTD and TMD (ΔDiCC = 0.105). Taken together, these results highlight the extent to which a single mutation in the SSD affects long-range communication in NPC1. The change in hydrogen bonding in the IBS is propagated through the entire protein, suggesting that the NPC1 protein relies on cross-talk between domains, and sterol transport is subject to these communication pathways.

**Figure 6:**
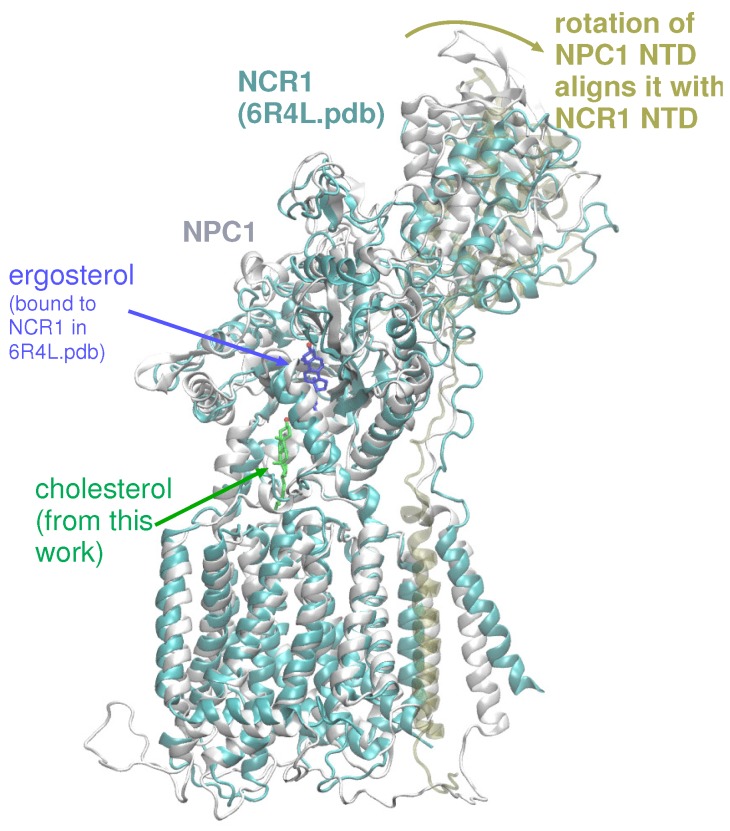
Superposition of NPC1, as modeled here (colored light grey) with cholesterol (green stick representation), superimposed on NCR1 (colored cyan) from PDB 6R4L, showing ergosterol (dark blue stick representation). A hinging of the NTD of NPC1 (colored tan) aligns it exactly with the NTD of NCR1, suggesting that this movement may be critical for sterol transport in mammalian NPC1 [13].

Based on our computations here and in a previous work [20], several putative cholesterol binding sites exist. In accordance with the tunnel prediction of Long et al. in the NPC1 structure (PDB ID 6UOX) [18], our tunnel calculations based on a ligand-free, WT NPC1 protein indicate a single tunnel, 162 Å in length, connecting the NTD domain and the SSD (Figure 3C); no tunnel has been identified that traverses the entire transmembrane domain. Winkler et al. recently published a similar tunnel in the yeast NPC protein, NCR1 [13]. Furthermore, the authors demonstrated ergosterol binding in the interior of the protein. The authors asserted that after cholesterol is transferred from NPC2 to the NTD of NCR1, a repositioning of the N-terminal domain allows for the sterol molecule to be transferred along the tunnel to the SSD. From the SSD, the sterol molecule can enter the outer leaflet of the lipid bilayer. Superposition of NPC1 and yeast NCR1 (Figure 6) shows the high degree of structural similarity between the eukaryotic proteins, as well as the similar binding poses of cholesterol (green) and ergosterol (blue) with the hydroxyl group pointing toward the N-terminal domain. A hinging of the NTD of NPC1 aligns it exactly with the NTD of NCR1, suggesting that this conformational change may be critical in the mammalian species for establishing the same transfer tunnel. In light of the conformational changes observed in the simulations of WT and P691S, such a transfer mechanism seems highly plausible for human NPC1.

Our simulations showed that cholesterol in P691S migrates along the putative tunnel. This migration in the mutant NPC1 could block the tunnel, thereby preventing cholesterol from being transferred in the opposite direction, namely from the lumenal to the cytoplasmic side, perhaps via the lipid bilayer. This blockage may explain the disrupted trafficking behavior observed in P691S [22,23]. One can also speculate how a blocked tunnel would impact cholesterol trafficking if NPC1 relies on neighboring NPC1 molecules to process cholesterol out of the lysosome. Indeed, recent experiments have suggested that NPC1 may act in tandem with one or more NPC1 molecules [25]. Depending on the nature of the NPC1 mutant (i.e., homozygous, compound heterozygous), a blocked transfer tunnel may have severe adverse trafficking effects.

## 3. Conclusions

Here, we modeled WT NPC1 and P691S with cholesterol located at the recently identified binding pose in the IBS. The 100 ns MD simulations were carried out for WT and P691S NPC1 in the solvated, lipid bilayer. In WT NPC1, cholesterol translocation was in the direction of the lipid bilayer, whereas in P691S, the sterol molecule moved along the putative transport tunnel in the direction of the lumenal domains. One critical structural factor responsible for these differences in dynamical behavior is the orientation of the side chain of Glu688. In WT, the side chain is oriented toward the hydroxyl group of cholesterol, while in P691S, Glu688 reorients to form a hydrogen bond network with Ser691, Tyr629, and Tyr1229 near the sterol’s isooctyl tail. In P691S, this hydrogen bond network, maintained over the course of the simulations, prevents the helices in the SSD from rearranging to allow cholesterol passage toward the lipid bilayer. Analyses of DiCC values indicated that the correlated domain motion changes when mutating Pro691 to Ser691, suggesting that long-range communication within the protein is responsible for sterol transport behavior. Disruption of correlated motion leads to changes in the dynamical behavior of cholesterol.

Cholesterol was modeled in the IBS with the hydroxyl group pointing towards the lumenal domains. This orientation was chosen as the insertion of cholesterol into the lipid bilayer would favor this relative orientation of the polar head-group. Nonetheless, our previous docking studies with cholesterol in WT NPC1 showed that cholesterol in the opposite orientation is also energetically stable [20], so future simulations should be carried out to check the dynamics of cholesterol-bound WT and P691S with the cholesterol’s isooctyl tail pointing toward the lumenal domains. We should emphasize that current structural evidence only supports cholesterol bound to the N-terminal domain of WT NPC1 [4]. Therefore, these simulations serve as tools to investigate the structural features that may affect the conformational behavior of both WT and mutant NPC1 proteins. The simulations carried out here are based on structural models that we have constructed from existing X-ray crystallography data (∼3 Å resolution) and cryo-EM data (∼4 Å resolution) (see Section 4 for details). The accuracy of the atomic positions in our models was therefore limited by the accuracy of diffraction data. Ongoing advancements in imaging technology, leading to improved resolution of diffraction data, will continue to reveal the mechanistic details of large membrane proteins such as NPC1.

Our results suggested that a lateral transfer of cholesterol from the IBS to the lipid bilayer may be one method of transporting cholesterol from the NPC1 protein out of the lysosome, namely via insertion into the lipid bilayer. What steps precede the delivery of cholesterol from the NTD to the IBS are as of now unknown. The transport of cholesterol may proceed via the putative tunnel that connects the NTD to the IBS, but alternative mechanisms, such as the delivery of the sterol by an additional molecule, such as a neighboring NPC1 molecule, cannot be ruled out. A worthwhile next investigation would be long (several hundred ns) MD simulations of NPC1 with cholesterol located in the binding pocket of the NTD, as has been experimentally observed in WT NPC1 [4]. This simulation would allow us to test whether conformational changes in NPC1 allow the sterol to travel along the putative tunnel from the NTD to the SSD. Furthermore, as three additional putative sterol docking sites were recently identified computationally [20], ongoing work will be directed at simulating the sterol-bound NPC1 to test the affect of ligand binding in these potential binding pockets. Together with contributions from structural biologists and biochemists, these simulations will continue to help elucidate the mechanistic details of cholesterol binding in NPC1, furthering the community’s understanding of lipid metabolism in the lysosome and advancing efforts to treat NPC disease.

## 4. Methods

### 4.1. Construction of the Model

The WT NPC1 protein was modeled according to the following steps. The protein atomic coordinates were constructed from two sets of structural data. The NTD (residues 23 to 288) was taken from the lower resolution (4.43 Å) cryo-EM PDB structure 3JD8 [6], and residues 334 to 1255 were taken from PDB structure 5U74 (3.33 Å from [7]). The two structures were overlapped using the Matchmaker tool in Chimera [26], which relies on the Needleman–Wunsch algorithm for finding the closest alignments between protein chains [27]. The new protein coordinates were saved, and any missing internal residues (289–333, 642–649, 800–813) were reconstructed using CHARMM [28]. The P691S mutant was constructed using CHARMM [28] to replace the atomic coordinates of proline at position 691 with those of serine; the geometry of the side chain atoms of S691 was then optimized with 500 steps of steepest descent (SD) energy minimization, followed by 1000 adopted basis Newton–Raphson (ABNR).

To determine an initial protonation pattern in NPC1, pKa values of all titratable residues were evaluated by electrostatic energy computations using the in-house software karlsberg+ [29,30,31]. This procedure, described in detail in our previous work [20], combines continuum electrostatics with structural relaxation of hydrogens and salt bridges. Assuming a lysosomal pH of ∼5, the following amino acid side-chains were thus protonated: His215, His441, His492, His510, His512, His758, His1016, His1029, His1170, Glu406, Glu688, and Glu742. All other amino acids were protonated according to standard protonation patterns using the H-build tool from CHARMM [28]. Finally, according to the crystal structures, the 15 known disulfide bonds were constructed using CHARMM, [Cys25-Cys74, Cys31-Cys42, Cys63-Cys109, Cys75-Cys113, Cys97-Cys238, Cys100-Cys160, Cys177-Cys184, Cys227-Cys243, Cys240-Cys247, Cys468-Cys479, Cys516-Cys533, Cys909-Cys914, Cys956-Cys1011, Cys957-Cys979, Cys967-Cys976].

**Figure 7:**
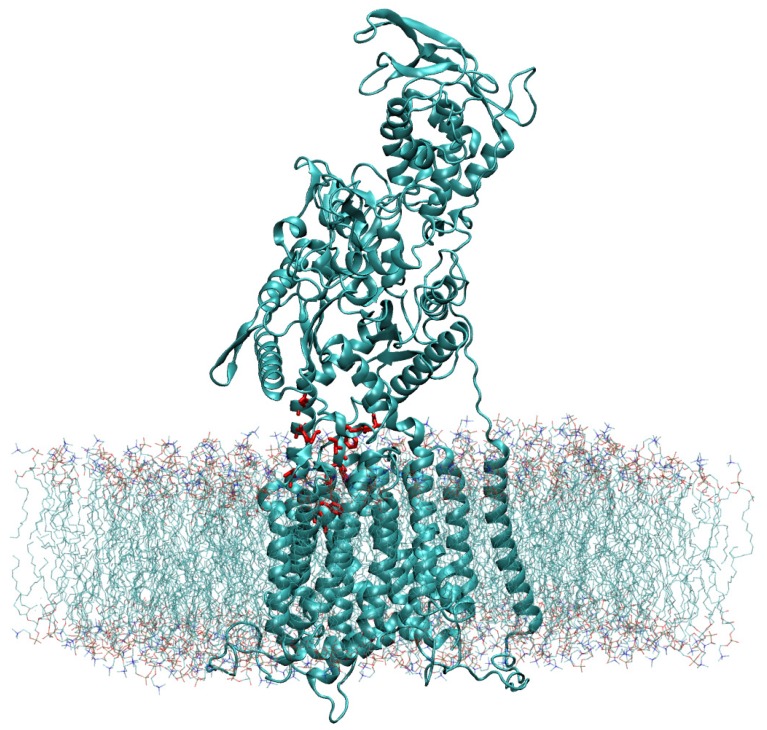
The NPC1 protein, modeled in a lipid bilayer according to the position as determined by the OPM database [34], is shown with the amino acid side chains of the IBS colored in red.

Using Chimera’s Matchmaker tool [26], we superimposed the constructed WT model with the recently resolved atomic model (PDB ID 6UOX [18]) that contains itraconazole bound to the NPC1 protein [18]. Next, the cholesterol molecule was modeled into the position of the itraconazole molecule with the hydroxyl group pointing “upward” toward the NTD. The coordinates of the cholesterol molecule, relative to the WT NPC1 protein, were saved. The cholesterol-bound protein was next modeled in the lipid bilayer using the CHARMM-GUI [32,33] and OPM database [34] (Figure 7).

For this, we assumed a lipid bilayer consisting of cholesterol (10%), DOPG (10%), and POPC (80%). The solvated protein in the lipid bilayer, including Na^+^ (196) and Cl^−^ (135) ions to neutralize charge and 73,919 explicit TIP3 water molecules [35], had a total size of 296,037 atoms and was simulated in a rectangular box of dimensions 126.8 Å × 126.8 Å × 195.0 Å.

The initial geometry of each solvated NPC1 model in the lipid bilayer was optimized with 10,000 steps of conjugate gradient energy minimization. As in our previous works [20,36], all energy minimizations and geometry optimizations used the all-atom CHARMM36 parameter set for the protein [37] and the TIP3P model for water molecules [35].

### 4.2. Molecular Dynamics Simulations

The solvated protein-membrane complex was simulated with molecular dynamics (MD) at 310 K according to the following protocol with NAMD [38]: (1) equilibration MD with Langevin dynamics (time step of 1 fs) for ∼400 ps; (2) equilibration MD with Langevin dynamics (time step of 2 fs) for 750 ps; production MD with Langevin dynamics (time step 2fs) for 100 ns. To simulate a continuous system, periodic boundary conditions were applied. Electrostatic interactions were summed with the Particle Mesh Ewald method [39] (grid spacing ∼1 Å; fftx 128, ffty 128, fftz 216). A nonbonded cutoff of 16.0 Å was used, and heuristic testing was performed at each energy call to evaluate whether the non-bonded pair list should be updated.

### 4.3. Calculation of Distance Correlation Coefficients

Distance correlation coefficients (DiCC) were calculated from the 100 ns MD trajectories using the dcor function in CHARMM [28,40]. DiCC, calculated from distance covariance, have been shown to best capture the correlation between positional vectors and to be a valid measure of concerted atomic motions as they are least sensitive to angular dependence [40,41]. For two vector series {A} and {B} containing the atomic positions from an MD trajectory, the DiCC between the two vectors is defined as:

(1) $$DiCC=\frac{\nu (A,B)}{\sqrt{\nu (A,A)\nu (b,B)}}$$

where ν(A,B) is the distance covariance between the vectors and is defined as:

(2) $$\nu \left(A,B\right)=\sqrt{\frac{1}{n^{2}}\sum^{ij}a_{ij}b_{ij}}.$$

The elements $a_{ij}=a_{ij}-a_{i}-a_{j}+a..$ are the elements of vector *A*, and *b_ij_* are analogously the elements of vector *B*. Using this approach, the motion of protein domains can be analyzed to evaluate the degree of long-range concerted motion, particularly in multi-domain proteins [41]. Here, the DiCC for each model were calculated from the MD trajectories by aligning first the protein backbone atoms and calculating the distance covariance for each pair of domains, based on the position of backbone atoms.

### 4.4. Tunnel Identification

Tunnel identification was carried out with MoleOnline [24]. Protein coordinates from the final snapshot of a 100 ns MD simulation of cholesterol-free WT NPC1 (model and simulation details given in [20]) were used for the calculation. The start position was set to Glu688, and the end position was Asn41 to obtain the 162 Å tunnel shown in Figure 3C.

## Figures and Tables

**Table 1:** Distance correlation coefficients (DiCC) for WT NPC1 and P691S and the changes in DiCC are listed. The domains are classified as follows: NTD (amino acids 30–250); MLD (amino acids 374–620); CTD (amino acids 861–1083); TM helices (amino acids 260–373, 621–797, and 1084–1278); linker (amino acids 251–259); cytosolic loops (aa 290–337) (see Figure 2). The strongest correlations in WT and P691S, as well as the largest differences in DiCC (ΔDiCC = |DiCC(WT) − DiCC(P691S)| are marked in bold.

	NTD	MLD	CTD	TMD Helices	Linker	Cyt Loops
**(A) WT**						
**NTD**	1.00	0.901	**0.935**	0.906	0.643	0.742
**MLD**	0.901	1.000	0.797	0.897	0.610	0.592
**CTD**	**0.935**	0.797	1.000	0.890	0.628	0.766
**TMD**	0.906	0.897	0.890	1.000	0.628	0.633
**linker**	0.643	0.610	0.628	0.628	1.000	0.470
**Cyt loops**	0.742	0.592	0.766	0.633	0.470	1.000
**(B) P691S**						
**NTD**	1.00	**0.912**	0.905	0.801	0.653	0.623
**MLD**	**0.912**	1.000	0.805	0.810	0.614	0.531
**CTD**	0.905	0.805	1.000	0.853	0.654	0.557
**TMD**	0.801	0.801	0.853	1.000	0.564	0.454
**linker**	0.653	0.614	0.654	0.564	1.000	0.514
**Cyt loops**	0.623	0.531	0.557	0.454	0.514	1.000
**(C)** |ΔDiCC|						
**NTD**	0.000	0.011	0.030	0.105	0.010	0.119
**MLD**	0.011	0.000	0.008	0.087	0.004	0.061
**CTD**	0.030	0.008	0.000	0.037	0.026	**0.209**
**TMD**	0.105	0.087	0.037	0.000	0.064	0.179
**linker**	0.010	0.004	0.026	0.064	0.000	0.044
**Cyt loops**	0.119	0.061	**0.209**	0.179	0.044	0.000

## Abbreviations

The following abbreviations are used in this manuscript:

Niemann–Pick Type C1 (NPC1), Niemann–Pick Type C2 (NPC2); amino acids (aa); N-terminal domain (NTD); middle lumenal domain (MLD); C-terminal domain (CTD); sterol-sensing domain (SSD); transmembrane (TM); itraconazole binding site (IBS); molecular dynamics (MD); distance correlation coefficient (DiCC).
